# Prurigo Pigmentosa: Dermoscopic Evaluation

**DOI:** 10.5826/dpc.1104a115

**Published:** 2021-10-01

**Authors:** Hitaishi Mehta, Anuradha Bishnoi, Keshavamurthy Vinay, Akanksha Kaushik, Muthu Sendhil Kumaran, Arpitha Kollabathula, Debajyoti Chatterjee, Uma Nahar Saikia, Davinder Parsad

**Affiliations:** 1 Department of Dermatology, Venereology and Leprology, Postgraduate Institute of Medical Education and Research, Chandigarh, India; 2 Department of Histopathology; Postgraduate Institute of Medical Education and Research, Chandigarh, India

**Keywords:** prurigo pigmentosa, keto rash, dermatoscopy, dermoscopy

## Introduction

Prurigo pigmentosa, also known as Nagashima’s disease or “keto rash”, is an uncommon inflammatory disorder of unknown etiology with a predilection for young Asian females. While erythematous papules, papulovesicles, and pruritus predominate in the acute stage, later lesions are characterized by pigmentary changes. Both stages might also present a reticulated pattern and frequently coexist. Dermoscopy is a non-invasive tool that allows for in-vivo detailed visualization of cutaneous lesions. The patterns seen on dermoscopic evaluation can also be correlated with histopathologic findings. Although several cases of prurigo pigmentosa have been reported in literature, data on dermatoscopy for this condition is scarce. We evaluated the dermoscopic patterns in a patient with prurigo pigmentosa. We conclude that these patterns correlate with histopathological findings.

## Case Presentation

**Figure 1:**
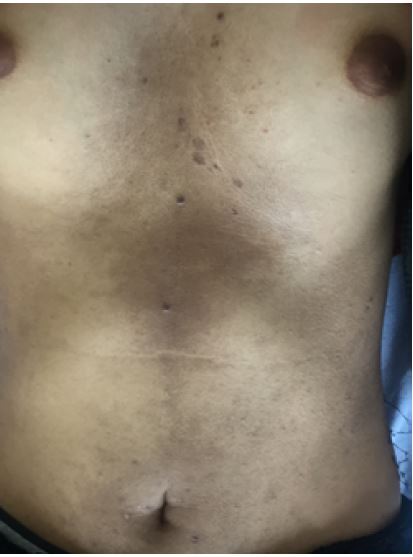
Clinical image of anterior trunk showing multiple papules surmounted on with macular pigmentation.

A 21-year-old woman sought dermatology consultation for a recurring rash over her trunk over the last 3 years. Individual lesions started as itchy, raised, and brownish bumps, which resolved over the next few days leaving brownish pigmentation. She had received topical corticosteroids and oral antihistamines in the past with minimal improvement. On examination of anterior trunk, the patient had multiple hyperpigmented papules overlying large brownish patches predominantly distributed in the intermammary and periumbilical distribution (Figure 1).

**Figure 2:**
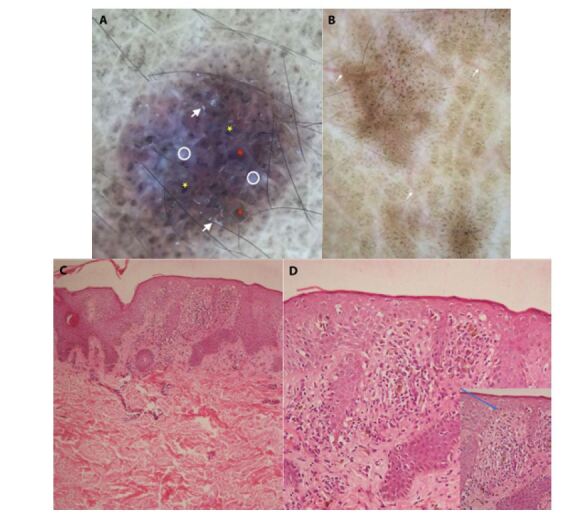
(A) Dermoscopic image of a fully developed lesion showing whitish scales (arrows), brown-red structureless areas with poorly defined borders (red stars), multiple irregularly distributed brownish blue-gray dots and globules (yellow stars), and blue-white veil like structures (white ovals) over a background of erythema (DermLite II, hybrid M, ×10, polarized).(B) Resolving lesions demonstrated brownish background reticular pigmentation surmounted by volcano-like islands composed of multiple irregular brown-gray dots and prominent linear blood vessels (white arrows, DermLite II, hybrid M, ×10, polarized). (C) Histopathology revealed mild hyperkeratosis, acanthosis, and superficial dermis showing perivascular inflammation on low power view. (D) Histopathology revealed basal cell vacuolization, pigment incontinence, apoptotic keratinocytes (arrow in inset), neutrophilic exocytosis, and interface dermatitis evident on high power magnification.

On dermatoscopy (DermLite II hybrid M dermatoscope, magnification ×10 in polarized noncontact mode equipped with Apple iPhone 6 plus camera), lesions in varying stages of evolution could be discerned. Papules revealed whitish scales, multiple irregularly distributed brownish black to blue-gray dots and globules, and blue-white veil-like structures over an ill-defined erythema background (Figure 2A). Resolving lesions demonstrated brownish background reticular pigmentation, islands of multiple irregular brown-gray dots sparing the skin creases, and prominent linear blood vessels (Figure 2B). Histopathology revealed mild hyperkeratosis, neutrophilic exocytosis, necrotic keratinocytes, interface dermatitis and pigment incontinence suggestive of prurigo pigmentosa (Figure 2, C and D). The patient was started on minocycline 100 mg once daily and showed resolution of erythematous pruritic papules (but not the hyperpigmentation) during a follow-up period of 2 months.

## Conclusion

Only one case report has described the dermoscopic findings of prurigo pigmentosa. Here we reported erythematous blanchable lesions in acute stage and grey spots at later stages [1]. Due to the darker skin tone of our patient, erythema was less perceivable; however, the dots and globules could be easily appreciated. Whitish scales in active lesions probably represented dyskeratosis, and blue-gray dots and globules represented extensive dermal pigment incontinence. The pattern of blood vessels could be seen in the resolving lesions in our patient and was linear rather than dotted.

Clinical differentials in our case included lichen planus, confluent and reticulated papillomatosis, primary cutaneous amyloidosis, Darier disease, Dowling-Degos disease, and frictional melanosis. The dermoscopic differentials have been summarized in Table 1. This case also shows resolving stages of all dermatoses characterized by interface dermatitis manifest blue gray dots and globules, albeit in different patterns and distributions; thus, making clinico-histologic and dermoscopic correlation mandatory [2].

We suggest the role of dermoscopy in providing support guiding diagnosis of prurigo pigmentosa, while acknowledging that these findings are based on the observation of a single patient. Further studies with larger sample size are suggested to support the findings presented here.

## Figures and Tables

**Table 1:** Various Differential Diagnosis and Their Dermoscopic Features

Differential diagnosis	Dermoscopic features
Lichen planus	Wickham striae in varying patterns with dotted, globular, or linear vessels at periphery of the lesion. Blue-gray dots in resolving stages
Confluent and reticulated papillomatosis	Polygonal, homogenous, brownish globules separated by whitish striae creating a cobblestone pattern along with fine whitish scaling
Frictional melanosis	Brownish structureless areas arranged in a reticular fashion
Acquired dermal macular hypermelanosis (ADMH)	Pigment dots, globules and blotches arranged in a dotted, chinese letter, hem-like, reticular, or diffuse pattern depending upon the severity of the disease [2]
Darier disease/Grover disease	Centrally located yellow-brown polygonal or star-shaped area surrounded by whitish halo, along with whitish scales and dotted or linear vessels, often with white halo [3]
Dowling-Degos disease	Irregular, star-shaped brownish outlines over a red–brown background with follicular plugging and inclusion cysts [4]
Prurigo pigmentosa (current case)	Whitish scales, brown-red structureless areas, irregularly distributed blue-gray dots and globules, and blue-white veil-like structures over a background of erythema
